# Integrating sleep-related breathing disorders and epigenetics into the genetic landscape of sleep disturbances in psychotic disorders

**DOI:** 10.1017/S0033291725100858

**Published:** 2025-06-26

**Authors:** Susana Perdigoto, Miguel De Pedro, Miguel Meira e Cruz

**Affiliations:** 1 Orofacial Pain, Oral Pathology and Dental Sleep Medicine Research Group. Department of Postgraduate Clinical Dentistry. Faculty of Biomedical Sciences. Universidad Europea de Madrid; 2 Sleep Unit, Centro Cardiovascular da Universidade de Lisboa (CCUL@RISE), Faculdade de Medicina de Lisboa, Lisboa, Portugal

**Keywords:** comorbid insomnia and sleep apnea, epigenetics, insomnia, sleep-disordered breathing, COMISA, obstructive sleep apnea, sleep apnea, psychotic disorders, psychiatric, sleep disturbances, psychosocial health, genetics

Recent advances in psychiatric genetics have underscored the role of polygenic scores (PGSs) in shaping sleep phenotypes in individuals with psychotic disorders. The study by Cederlöf et al. (2025) makes an important contribution by leveraging genome-wide association data to disentangle the complex interplay between genetic liability for insomnia, sleep duration, chronotype, and schizophrenia. Their findings offer compelling insights into how distinct genetic risks manifest in both subjective experiences and cognitive performance. Yet, the genetic landscape of sleep in psychosis is even more nuanced than the current framework suggests. Several biologically and clinically relevant dimensions—namely sleep-related breathing disorders (SRBDs), the COMISA phenotype (comorbid insomnia and sleep apnea), and the dynamic influence of epigenetic regulation—warrant deeper integration into this conversation.

Obstructive sleep apnea (OSA), the most prevalent SRBD, is significantly underrecognized in psychotic populations despite high clinical relevance. Individuals with schizophrenia are at increased risk for OSA due to overlapping predisposing factors, including antipsychotic-induced weight gain, sedative use, metabolic syndrome, and lifestyle-related variables (Annamalai et al., 2015; Giles et al., 2022). The consequences of untreated OSA—fragmented sleep, intermittent hypoxia, and excessive daytime sleepiness—may mimic, mask, or exacerbate core psychiatric symptoms, such as cognitive impairment and mood instability (Knechtle et al., 2019). Importantly, studies have shown that treatment with continuous positive airway pressure (CPAP) improves psychiatric outcomes, including mood and attention, in individuals with psychotic disorders (Giles et al., 2022). Despite this, genetic models of sleep in psychosis rarely incorporate SRBDs or the emerging polygenic scores for OSA recently developed from population-based cohorts (Liu et al., 2024). Their absence may obscure an important dimension of biological vulnerability.

The intersection of insomnia and OSA—referred to as COMISA—represents a distinct and consequential phenotype. Epidemiological data suggest that COMISA is more prevalent than either disorder alone and associated with more severe clinical outcomes, including greater psychiatric symptom burden, metabolic dysregulation, and treatment resistance (Meira, Cruz, & Sweetman, 2025). The co-occurrence of insomnia and OSA creates a synergistic burden through the convergence of hyperarousal, sleep fragmentation, oxidative stress, and HPA axis dysregulation. These mechanisms overlap with known neurobiological signatures of psychosis, including circadian misalignment, impaired slow-wave sleep, and abnormal synaptic plasticity. Importantly, COMISA complicates therapeutic response, as isolated treatments, such as CBT-I or CPAP, often fail unless the dual pathology is addressed comprehensively. It is plausible that COMISA constitutes not just a clinical entity but a genetic and epigenetic endophenotype of psychosis, deserving focused investigation. The lack of consideration of COMISA in Cederlöf et al.’s study may therefore limit the scope of their otherwise valuable findings.

While PGSs illuminate inherited predispositions, they do not capture the environmentally responsive layer of gene regulation provided by epigenetics. Mechanisms such as DNA methylation, histone acetylation, and non-coding RNAs mediate the interface between genotype and environment, dynamically shaping gene expression in response to stressors, including sleep loss (Gaine, Chatterjee, & Abel, 2018), hypoxia (Verdikt & Thienpont, 2024), psychosocial stress (Wu, Qu, Zhang, & Liu, 2024), and pharmacological exposure (Werner, Altshuler, Shaham, & Li, 2021). Epigenetic abnormalities have been widely observed in schizophrenia, affecting genes involved in synaptic signaling, neurodevelopment, and circadian regulation (Yang, Sun, Li, & Zhang, 2025). Sleep disorders, such as OSA and insomnia, exert measurable epigenetic effects. Sleep fragmentation in OSA alters methylation in genes related to inflammation and neuronal excitability (Leader et al., 2021), while intermittent hypoxia induces region-specific histone modifications in brain regions central to cognition and psychosis, including the hippocampus and prefrontal cortex (Ou, Zong, & Ouyang, 2023). Likewise, chronic insomnia, through sustained activation of stress-related systems, may reinforce maladaptive methylation patterns (Palagini et al., 2023).

This growing body of evidence supports a shift from single-layer genetic analysis toward a comprehensive multi-omic framework that integrates genomics, epigenomics, transcriptomics, and high-resolution phenotypic data (Zhang et al., 2020). Objective sleep metrics—polysomnography, actigraphy, respiratory parameters, and circadian phase markers—are essential to adequately capture the complexity of sleep phenotypes in psychosis. Reliance on self-report measures, though convenient, may compromise accuracy, particularly in populations where cognitive and metacognitive deficits are common. Future studies should consider incorporating composite PGSs for OSA, COMISA, and chronobiological misalignment to refine predictive models.

These insights are not merely theoretical. They have substantial implications for clinical care. Patients with high genetic liability for insomnia but no SRBDs may benefit most from behavioral therapies like CBT-I. Conversely, individuals with overlapping risk for both insomnia and OSA—particularly those with COMISA—require a dual-pronged approach, combining behavioral, mechanical, and possibly pharmacological treatments. COMISA in psychosis may signify a subgroup with elevated risk for cognitive decline, functional deterioration, and treatment non-response, thereby meriting targeted early intervention. Moreover, recognition of the epigenetic plasticity of sleep-related pathways introduces new possibilities for preemptive and therapeutic modulation, as epigenetic changes induced by sleep disruption are reversible with adequate intervention. CBT-I, for example, has been shown to normalize stress-related gene methylation, while CPAP use may reverse hypoxia-induced epigenetic marks.

In summary, while the study by Cederlöf et al. marks an important advance in understanding sleep-related polygenic influences in psychosis, we argue for a broader conceptualization that incorporates sleep-related breathing disorders, the COMISA phenotype, and the critical role of epigenetic regulation. A deeper integration of these dimensions may allow for more accurate stratification of sleep phenotypes, refinement of risk prediction models, and ultimately, the development of targeted interventions that improve outcomes for individuals with psychotic disorders.

## References

[r1] Annamalai, A., Palmese, L. B., Chwastiak, L. A., Srihari, V. H., & Tek, C. (2015). High rates of obstructive sleep apnea symptoms among patients with schizophrenia. Psychosomatics, 56(1), 59–66. 10.1016/j.psym.2014.02.009 25023923 PMC5583522

[r2] Cederlöf, E., Holm, M., Kämpe, A., Ahola-Olli, A., Kantojärvi, K., Lähteenvuo, M., … Paunio, T. (2025). Sleep and schizophrenia polygenic scores in non-affective and affective psychotic disorders. Psychological Medicine, 55, e117. 10.1017/S0033291725000844 40230302 PMC12094621

[r3] Gaine, M. E., Chatterjee, S., & Abel, T. (2018). Sleep deprivation and the epigenome. Frontiers in Neural Circuits, 12, 14. 10.3389/fncir.2018.00014 29535611 PMC5835037

[r4] Giles, J. J., Ling, I., McArdle, N., Bucks, R. S., Cadby, G., Singh, B., … Waters, F. (2022). Obstructive sleep apnea is treatable with continuous positive airway pressure in people with schizophrenia and other psychotic disorders. Schizophrenia Bulletin, 48(2), 437–446. 10.1093/schbul/sbab100 34581411 PMC8886585

[r5] Knechtle, B., Economou, N. T., Nikolaidis, P. T., Velentza, L., Kallianos, A., Steiropoulos, P., … Trakada, G. (2019). Clinical characteristics of obstructive sleep apnea in psychiatric disease. Journal of Clinical Medicine, 8(4), 534. 10.3390/jcm8040534 31003451 PMC6518048

[r6] Leader, B. A., Koritala, B. S. C., Moore, C. A., Grigg Dean, E. H., Kottyan, L. C., & Smith, D. F. (2021). Epigenetics of obstructive sleep apnea syndrome: A systematic review. Journal of Clinical Sleep Medicine, 17(12), 2533–2541. 10.5664/jcsm.9514 34176557 PMC8726381

[r7] Liu, H., Wang, X., Feng, H., Zhou, S., Pan, J., Ouyang, C., & Hu, X. (2024). Obstructive sleep apnea and mental disorders: A bidirectional Mendelian randomization study. BMC Psychiatry, 24(1), 304. 10.1186/s12888-024-05754-8 38654235 PMC11040841

[r8] Meira, e., Cruz, M., & Sweetman, A. (2025). Comorbid insomnia and sleep apnea (COMISA): From research to clinical practice. Seminars in Respiratory and Critical Care Medicine. Advance online publication. 10.1055/a-2591-5664 40258387

[r9] Ou, Y., Zong, D., & Ouyang, R. (2023). Role of epigenetic abnormalities and intervention in obstructive sleep apnea target organs. Chinese Medical Journal, 136(6), 631–644. 10.1097/CM9.0000000000002080 35245923 PMC10129098

[r10] Palagini, L., Geoffroy, P. A., Gehrman, P. R., Miniati, M., Gemignani, A., & Riemann, D. (2023). Potential genetic and epigenetic mechanisms in insomnia: A systematic review. Journal of Sleep Research, 32(6), e13868. 10.1111/jsr.13868 36918298

[r11] Verdikt, R., & Thienpont, B. (2024). Epigenetic remodelling under hypoxia. Seminars in Cancer Biology, 98, 1–10. 10.1016/j.semcancer.2023.10.005 38029868

[r12] Werner, C. T., Altshuler, R. D., Shaham, Y., & Li, X. (2021). Epigenetic mechanisms in drug relapse. Biological Psychiatry, 89(4), 331–338. 10.1016/j.biopsych.2020.08.005 33066961 PMC7854851

[r13] Wu, Z., Qu, J., Zhang, W., & Liu, G. H. (2024). Stress, epigenetics, and aging: Unraveling the intricate crosstalk. Molecular Cell, 84(1), 34–54. 10.1016/j.molcel.2023.10.006 37963471

[r14] Yang, H., Sun, W., Li, J., & Zhang, X. (2025). Epigenetic factors in schizophrenia: Future directions for etiologic and therapeutic study approaches. Annals of General Psychiatry, 24(1), 21. 10.1186/s12991-025-00557-x 40186258 PMC11969811

[r15] Zhang, L., Lu, Q., & Chang, C. (2020). Epigenetics in health and disease. Advances in Experimental Medicine and Biology, 1253, 3–55. 10.1007/978-981-15-3449-2_1 32445090

